# HIV infection of thymocytes inhibits IL-7 activity without altering CD127 expression

**DOI:** 10.1186/1742-4690-8-72

**Published:** 2011-09-16

**Authors:** Charlene D Young, Jonathan B Angel

**Affiliations:** 1 Ottawa Hospital Research Institute, 501 Smyth Rd., Ottawa, Canada; 2 Department of Biochemistry, Microbiology and Immunology, University of Ottawa, 450 Smyth Rd., Ottawa, Canada; 3 Division of Infectious Diseases, Ottawa Hospital-General Campus, 501 Smyth Rd.Ottawa, Canada

## Abstract

**Background:**

Thymic function is altered in HIV infection and characterized by dysregulation of the thymic epithelial network, reduced thymic output and ultimately an impaired naïve T-cell pool. The IL-7/IL-7 receptor (IL-7R) signalling pathway is critical for the maturation and differentiation of thymocytes. HIV infection is associated with a decrease in IL-7Rα (CD127) expression and impaired CD127 signalling in circulating CD8^+ ^T-cells; however, little is known about the effect of HIV on CD127 expression and IL-7 activity in the thymus. Therefore, the effect of *in vitro *HIV infection on CD127 expression and IL-7-mediated function in thymocytes was investigated.

**Findings:**

*In vitro *HIV infection of thymocytes did not affect CD127 expression on either total thymocytes or on single positive CD4 or single positive CD8 subsets. However, HIV infection resulted in a decrease in the level of IL-7-induced STAT-5 phosphorylation and Bcl-2 expression in unfractionated thymocytes.

**Conclusion:**

These findings indicate that HIV infection alters IL-7 responsiveness of thymocytes by a mechanism other than CD127 downregulation and potentially explain the disruption in thymopoiesis observed in HIV infection.

## Findings

Human immunodeficiency virus (HIV) infection is characterized by a loss of CD4^+ ^T-cells and a progressive loss in cytotoxic T-cell lymphocyte (CTL) function resulting in immunodeficiency. HIV infection has also been associated with impaired thymic output [1]. Examination of the thymus of HIV-infected pediatric patients reveals selective thymocyte depletion and disruption of the thymic microenvironment, which is thought to contribute to more rapid progression to AIDS [2-4]. In HIV-1 infected SCID-hu Thy/Liv mouse models, there is a depletion of intrathymic progenitor T-cells which precedes the loss of infected CD4^+^CD8^+ ^thymocytes, suggesting that HIV infection interrupts thymocyte development at an early stage [5]. However, the mechanisms of disrupted thymic development by HIV have yet to be fully elucidated.

Interleukin-7 (IL-7) is a pleiotropic cytokine that is critical for several stages of thymopoiesis, maintains mature T-cell homeostasis, enhances CTL function and increases T-cell survival [6-14]. IL-7 signals through the IL-7 receptor complex (IL-7R), which is composed of two subunits: the IL-7Rα chain (CD127), that is also shared by TSLP [15], and the IL-2Rγ chain which is shared by a number of other cytokines including IL-2, IL-4, IL-9, IL-15 and IL-21 [7,8]. The role of IL-7 in thymopoiesis is multifaceted, as it is critical for early stages of T-cell development in allowing chromatin accessibility to enable T-cell receptor VDJ gene rearrangement, inducing thymocyte proliferation and maintaining thymocyte survival by upregulating the anti-apoptotic protein Bcl-2 and downregulating the pro-apoptotic protein Bax [12,16-18]. Disrupting IL-7 signalling can result in profoundly impaired immunity as seen in patients with T^-^B^+^NK^+ ^Severe Combined Immunodeficiency (SCID), a genetic defect that results in inactivation of the IL-7Rα signalling pathway [19]. The importance of the IL-7 signalling complex in thymic development was confirmed in knock-out mice for both IL-7 and IL-7R. IL-7^-/- ^mice have a 20 fold decrease in thymic cellularity and an increase in triple negative (TN) cells, indicative of a developmental block at the TN stage [20]. The phenotype with IL-7R^-/- ^knockout mice is much more severe with a 90-99.99% decrease in thymic cellularity [13].

We and others have previously demonstrated that HIV infection is associated with decreased CD127 expression on circulating CD8^+ ^T-cells, and with effective antiretroviral therapy CD127 expression on T-cells is partially restored [21-23]. The regulation of CD127 by HIV may play a role in disease pathogenesis since the expression of CD127 has been correlated with measures of disease progression (decreased CD4 count, increased viral load, increased immune activation) [24,25]. In addition to decreased CD127 expression on T-cells, we and others have also shown that CD127 signalling is impaired in HIV infection [26-28]. Given the importance of the role of IL-7 in HIV pathogenesis and the current development of IL-7 as a therapeutic agent for HIV infection and other conditions, understanding the mechanism by which HIV impairs IL-7 activity within the thymus is of the greatest importance. The aim of this study is to evaluate the effects of HIV infection on CD127 expression and IL-7 activity in primary human thymocytes.

**Figure 1:**
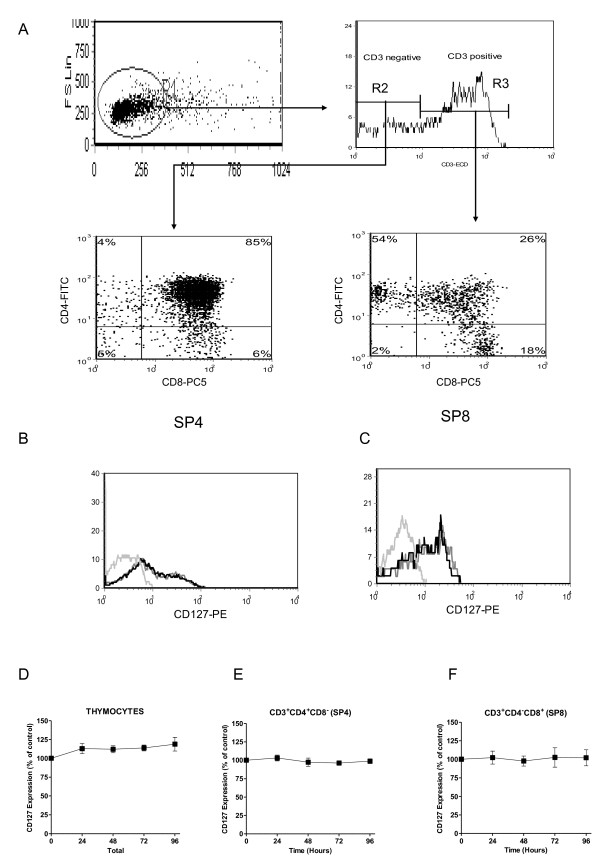
**HIV infection does not alter CD127 expression on thymocytes**. A) Unfractionated thymocytes (gate 1) were identified based on the forward scatter/side scatter profiles of live cells. The cells were then gated on either CD3**^- ^**(gate 2) or CD3**^+ ^**(gate 3) in a single parameter histogram. The cells in gate 2 and gate 3 were then analysed for CD4 and CD8 expression. The expression of CD127-PE (Beckman Coulter) was measured on the various subsets. Thymocytes were incubated with HIV**_cs204 _**or mock infected and co-cultured with thymic epithelial cells for up to 96 hours. Light grey lines represent isotype control, mock infected (black line) and HIV infected (dark grey line). B)SP4 subset C)SP8 subset. Summary data of CD127 expression on thymocyte measured as the proportion of cells expressing CD127 relative to mock infected cultures on D) unfractionated thymocytes, E) SP4, and F) SP8 thymic subsets.

It has been widely reported that CD127 expression is decreased on circulating CD4^+ ^and CD8^+ ^T-cells of HIV-infected individuals [21-25]. We have also recently shown that *in vitro *infection of peripheral blood mononuclear cells (PBMC) results in decreased CD127 expression on CD8^+ ^T-cells [29]. We therefore investigated the effect of *in vitro *HIV infection on CD127 expression on thymocytes as a potential mechanism of HIV-induced thymic dysfunction. Thymocytes were infected *in vitro *with a primary isolate cs204 following previously described methods [30,31]. Briefly unfractionated thymocytes were treated with polybrene (3 μg/ml) (Sigma-Aldrich., Oakville, Ont) for one hour prior to infection with the dual tropic strain HIV_cs204 _at an M.O.I. of 0.01 or mock-infected with equivalent volumes of PBMC culture supernatants. Two hours post infection (p.i), cells were washed in phosphate buffer saline (PBS) (Invitrogen, Burlington, On), resuspended to 1.0 × 10^6^/ml and co-cultured with thymic epithelial cells (TEC) (1:25) for up to 96 hours. CD127 expression on thymocytes was analysed every 24 h by flow cytometry. Thymocytes were stained with the following fluorochrome-conjugated monoclonal antibodies: CD3-ECD (clone UCHT1), CD4-FITC (clone 13B8.2), CD8-PC5 (clone B9.11), CD127-PE (clone R34.34) (all from Beckman Coulter). The distribution of the following developmental stages of T-cell maturation was evaluated: (TN) CD3-CD4-CD8-, (immature single positive CD4 ISP4+) CD3-CD4+CD8-, (DP) CD3+/-CD4+CD8+ and (SP) CD4+ or CD8+ cells. The gating strategy for phenotype analysis is depicted in Figure 1A. There was no change in CD127 expression on unfractionated thymocytes following HIV infection over 96 hours in culture (Figure 1D). Although HIV infection did not alter CD127 expression in unfractionated thymocytes, a specific effect on individual thymic subsets may have been masked. We, therefore, infected total thymocytes and measured CD127 expression on individual thymic subsets by flow cytometry. The thymocyte subset distribution within the culture system remained unchanged over a 96 hour culture period regardless of HIV infection (data not shown). *In vitro *HIV_cs204 _infection did not alter CD127 expression on immature thymic subsets (i.e. TN, ISP4 and DP subsets; data not shown) or on the more mature single positive CD4^+ ^(SP4) or single positive CD8^+ ^(SP8) thymocytes (Figure 1B-C).

**Figure 2:**
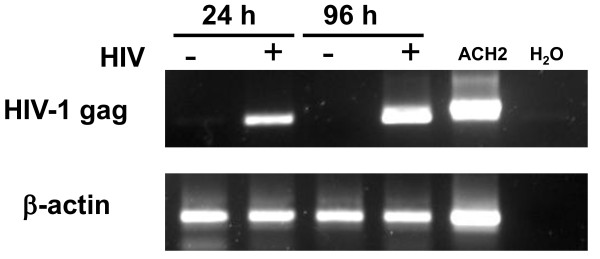
**Thymocytes are infected by HIV**. Thymocytes were incubated with HIV_cs204_, or mock infected and co-cultured with thymic epithelial cells. DNA was isolated from the cells following 24 hours or 96 hours p.i and the presence of HIV-1 was measured by nested PCR. As a positive control, DNA was isolated from ACH2 cells, and water was used as a negative control in the PCR reaction. Results are representative of 3 separate experiments.

To confirm HIV infection of thymocytes, genomic DNA was isolated from infected thymocytes as early as 24 hours and up to 96 hours p.i. Viral DNA was detectable by nested PCR targeting the gag region of HIV (Figure 2). Briefly, genomic DNA was isolated from infected thymocytes using the QIAGEN DNeasy blood and tissue kit (Qiagen, Mississauga, ON,). In the first round of PCR, DNA (1/10) was amplified with outer P24 primers (400 nm) forward (fwd): 5'-ATAGAGGAAGAGCAAAACAAAA-3'; reverse (rvs): 5'-GTTCCTGAAGGGTACTAGTAGT-3'. The second round PCR used 5 μl of the product from the first round of PCR with inner p24 primers (400 nm) fwd 5'-CAAAATTACCCTATAGTGCA-3' and rvs 5'-ATGTCACTTCCCCTTGGTTCT-3'. Amplification conditions were as follows: 2 minutes at 95°C, (94°C for 60 s, 55°C for 60 s and 72°C for 60 s) for 30 cycles and 7 minutes at 72°C.

**Figure 3:**
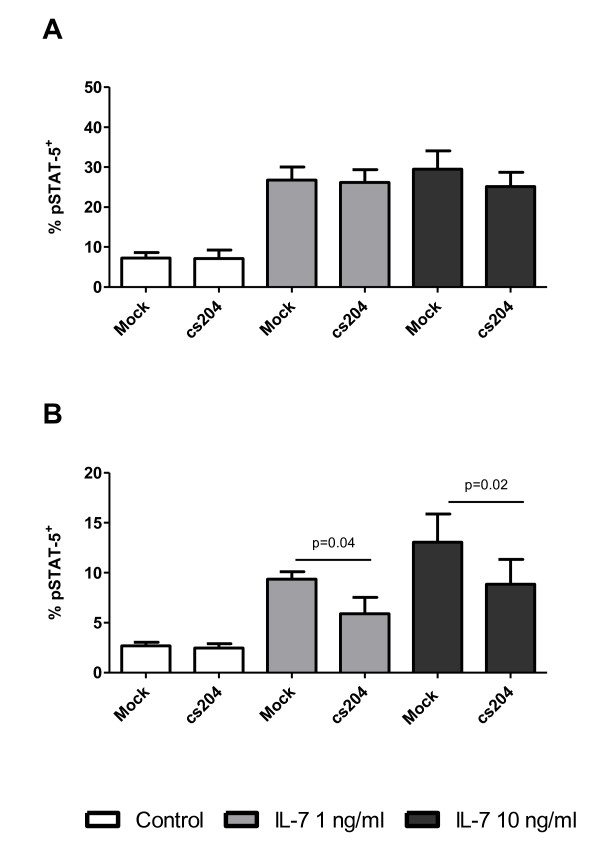
**The effect of *in vitro *HIV infection on IL-7-induced STAT-5 phosphorylation in thymocytes**. Thymocytes were incubated with HIV_cs204 _or mock infected and co-cultured with thymic epithelial cells. Following co-culture for A) 24 hours or B) 96 hours, thymocytes were stimulated with IL-7, and STAT-5 phosphorylation in the total thymocyte population was measured by intracellular flow cytometry.

While *in vitro *HIV infection did not affect surface CD127 expression on thymocytes, it remains possible that *in vitro *HIV infection is associated with altered IL-7 signalling as has been reported in CD8^+ ^T-cells from HIV-infected individuals [26-28]. This was, therefore, evaluated by measuring IL-7 responsiveness of thymocytes following HIV infection. Thymocytes were infected as described above, co-cultured with TEC for up to 96 hours and stimulated with IL-7 (1 ng/ml) (Sigma-Aldrich Inc., Oakville Ont) (0-10 ng/ml) for 15 minutes as previously described [32]. Cells were then fixed, permeabilized, stained with Alexa Fluor^® ^488 mouse antihuman STAT5 pY694 (BD Biosciences, San Jose, CA, USA) and analysed by flow cytometry. Thymocytes were cultured with HIV for 24 hours in order to allow sufficient time to establish infection. HIV had no impact on IL-7-induced pSTAT-5 expression when evaluated 24 p.i. (Figure 3A). However, thymocytes that were infected with HIV and cultured for longer periods of time (96 hours) had lower levels of IL-7-induced pSTAT-5 compared to mock-infected controls (Figure 3B). The change in the level of STAT-5 phosphorylation was not due to changes in cell viability, since there was no significant difference in viability between HIV infected and mock infected cultures after 96 hours of culture (data not shown).

**Figure 4:**
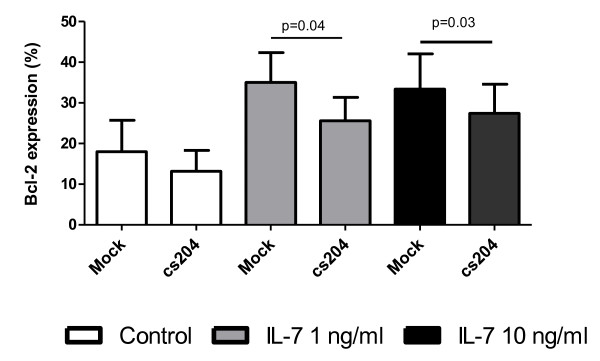
**The effect of HIV infection on the ability of IL-7 to induced Bcl-2 expression in thymocytes**. Thymocytes were incubated with HIV***_cs204 _***or mock infected and co-cultured with thymic epithelial cells for 24 hours. After 24 hours of culture, thymocytes were stimulated with IL-7 for 48 hours, and Bcl-2 expression was measured by intracellular flow cytometry.

IL-7 signalling is known induce Bcl-2 expression in thymocytes [33]. In order to further determine if *in vitro *HIV infection alters IL-7 function, the level of IL-7-induced Bcl-2 expression in HIV-infected thymocyte cultures was measured. Twenty-four hours p.i., cells were washed and stimulated with IL-7 (0-10 ng/ml) for 48 hours as previously established for optimal Bcl-2 induction by IL-7 [34]. Cells were then fixed, permeabilized, stained with Bcl-2-FITC (BD Bioscience) and analysed by flow cytometry. As expected, 48 hours of stimulation with IL-7 resulted in increased Bcl-2 expression in unfractionated thymocytes. *In vitro *HIV infection resulted in a small but non-significant decrease of constitutive Bcl-2 expression. Consistent with what was seen with the effect on STAT-5 activation, infection with HIV_cs204 _inhibited the ability of IL-7 to induce Bcl-2 expression in thymocytes (Figure 4).

IL-7 also signals through the PI3K pathway leading to cell proliferation and glucose uptake [35]. Thymocytes were infected for up to 96 hours, serum starved for 2 hours and stimulated with IL-7 (10 ng/ml) for 1 hour. Cells were lysed, and proteins were separated on an 8% SDS-polyacrylamide gel and transferred to a nitrocellulose membrane. Activation of the PI3K pathway was visualised by probing the membranes with antibodies for phosphorylated AKT (Cell Signalling, Danvers, MA). In contrast to its effect on STAT-5 and Bcl-2, HIV infection did not affect the ability of IL-7 to induce PI3K posphorylation (data not shown).

The importance of IL-7 and its effect on thymopoiesis are unequivocal. Disrupting this pathway leads to a block in thymopoiesis and the arrest of T-cell development. IL-7 signals through both the JAK/STAT and PI3K pathways to mediate cell survival, proliferation and differentiation [35,36]. HIV infection both *in vitro *and *in vivo *is associated with reduced CD127 on CD4^+ ^T-cells and CD8^+ ^T cells [21-25]. We have, however, demonstrated that *in vitro *HIV infection of thymocytes does not affect the surface expression of CD127 on thymocytes. The decreased CD127 expression on CD8^+ ^T-cells following *in vitro *HIV infection appears to be due to soluble factors released in the culture microenvironment by PBMCs [29]. Any such factors present in PBMC cultures may not be present in thymocyte/TEC co-cultures, potentially accounting for the differential effect of HIV on CD127 expression.

Although decreased IL-7 activity can result from decreased receptor expression, a block in the IL-7 signalling pathway may also result in altered IL-7 activity. This phenomenon has been reported for IL-2 activity where CD4^+ ^T-cells and CD8^+ ^T-cells from HIV^+ ^individuals are less responsive to IL-2 compared to those from healthy controls which has been attributed to a block in the JAK/STAT pathway [37,38]. The results in this report indicate that IL-7-induced STAT-5 phosphorylation and Bcl-2 expression are impaired in thymocyte cultures infected with HIV_cs204 _while no effect on CD127 expression was observed. This suggests that HIV infection results in a block in the IL-7 pathway that occurs independent of its effect on CD127 expression. These data support the findings by Vranjkovic *et al*., which demonstrated reduced IL-7 responsiveness in CD127-expressing CD8^+ ^T-cells from HIV^+ ^patients. In that study, isolated CD8^+^CD127^+ ^cells from HIV^+ ^individuals had lower levels of STAT-5 phosphorylation following IL-7 stimulation when compared to those from uninfected controls [26]. Such a block in IL-7 signalling has also been observed in other disease states. For example, CD4^+ ^and CD8^+ ^T-cells isolated from breast cancer patients are less responsive to IL-7, as measured by STAT-5 phosphorylation [39].

HIV may affect thymocyte function by altering the viability of the cells, consequently lowering the output of functional T-cells from the thymus [2,3,40,41]. In support of this hypothesis, our data show that HIV infection interferes with the ability of IL-7 to induce Bcl-2 expression. A similar block in the ability of IL-7 to upregulate Bcl-2 expression was reported in a study in which CD4^+ ^T-cells from HIV^+ ^individuals had lower levels of Bcl-2 expression following IL-7 stimulation when compared to those from healthy controls. That study found no correlation between CD127 expression of CD4^+ ^T cells and IL-7 responsiveness, suggesting that the block in IL-7 activity was independent of the level of CD127 expression [42].

The exact mechanism by which HIV interferes with the IL-7 signalling pathway has yet to be determined, however our results indicate that binding of HIV to the cell surface is likely insufficient to mediate this effect since there was no impact of HIV on IL-7 activity within the first 24 hours of infection. Rather, our data demonstrated that the cells need to be infected for longer periods of time (72-96 hours) for the effect of HIV to be observed, suggesting that the mechanism of inhibition might require the production of specific cellular or viral factors.

In summary, we demonstrated that HIV infection alters IL-7 activity in thymocytes independent of CD127 expression suggesting a potential mechanism by which HIV infection interrupts thymic output and contributes to immune deficiency.

## Competing interests

The authors declare that they have no competing interests.

## Authors' contributions

CY participated in the design of the study, performed the experiments and wrote the manuscript. JA conceived of the study, participated in the design of the study and helped to draft and edit the manuscript.
